# Supplementary figures and images for: Identification of Causal Relationships between Gut Microbiota and Influenza a Virus Infection in Chinese by Mendelian Randomization
Source: Microorganisms. 2024 Jun 8;12(6):1170. doi: 10.3390/microorganisms12061170 (PMC11205835; doi:10.3390/microorganisms12061170)

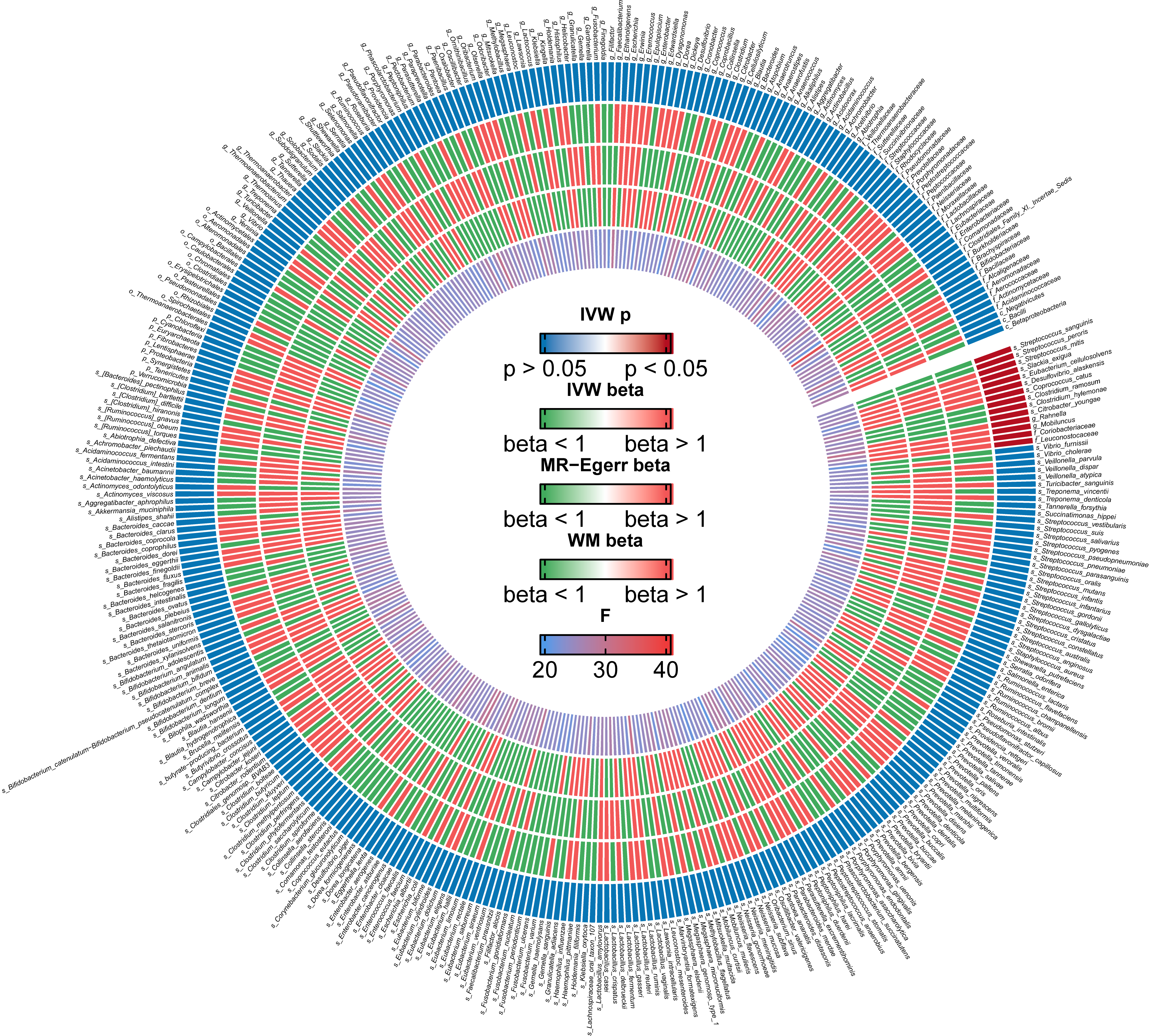

Supplement: Supplementary file 1 [file microorganisms-12-01170-s001.zip › Figure S1.tif]

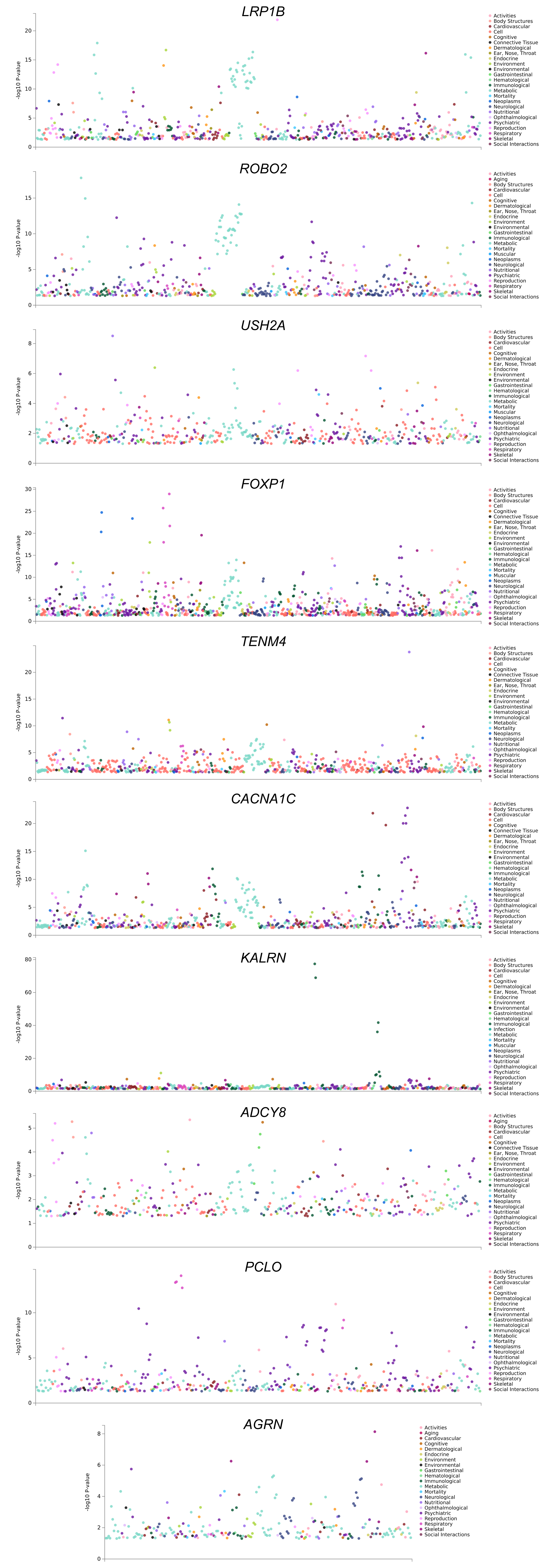

Supplement: Supplementary file 1 [file microorganisms-12-01170-s001.zip › Figure S10.tif]

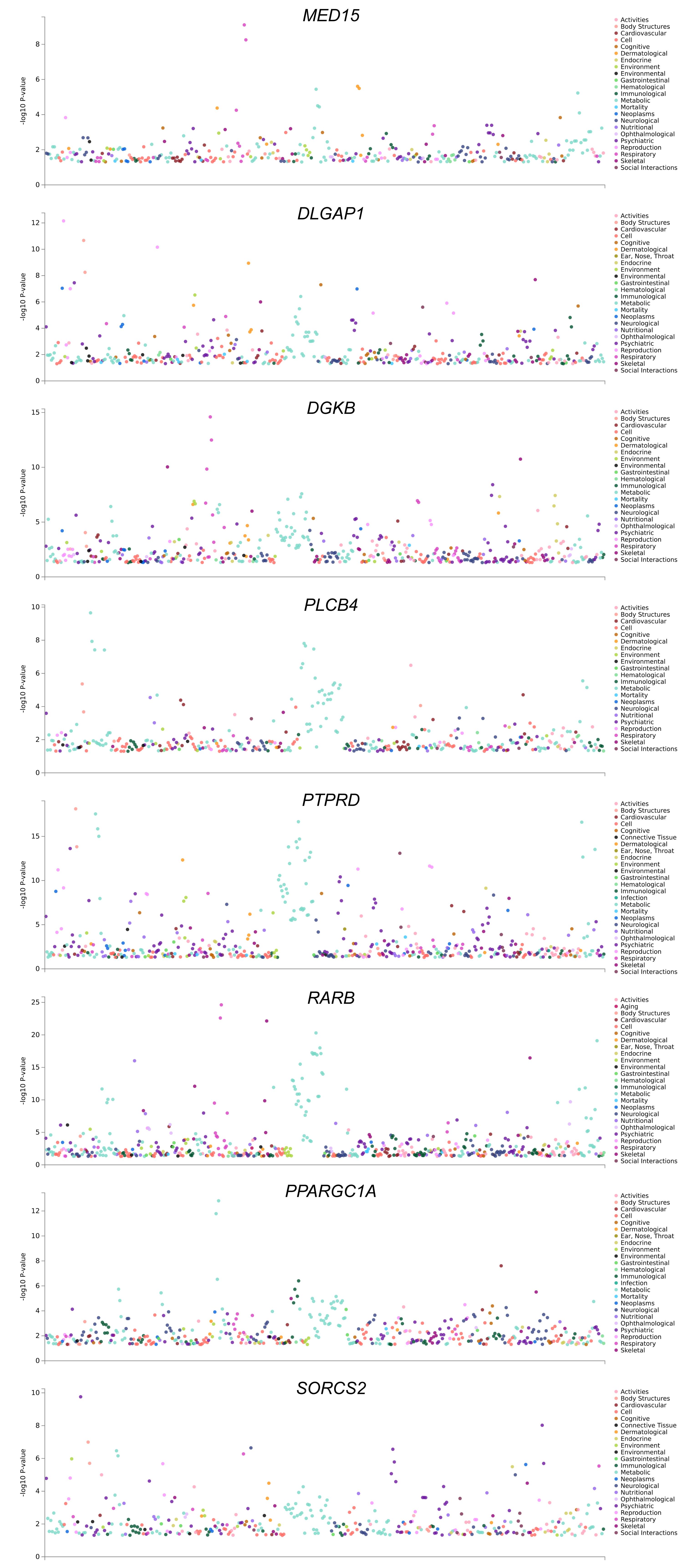

Supplement: Supplementary file 1 [file microorganisms-12-01170-s001.zip › Figure S11.tif]

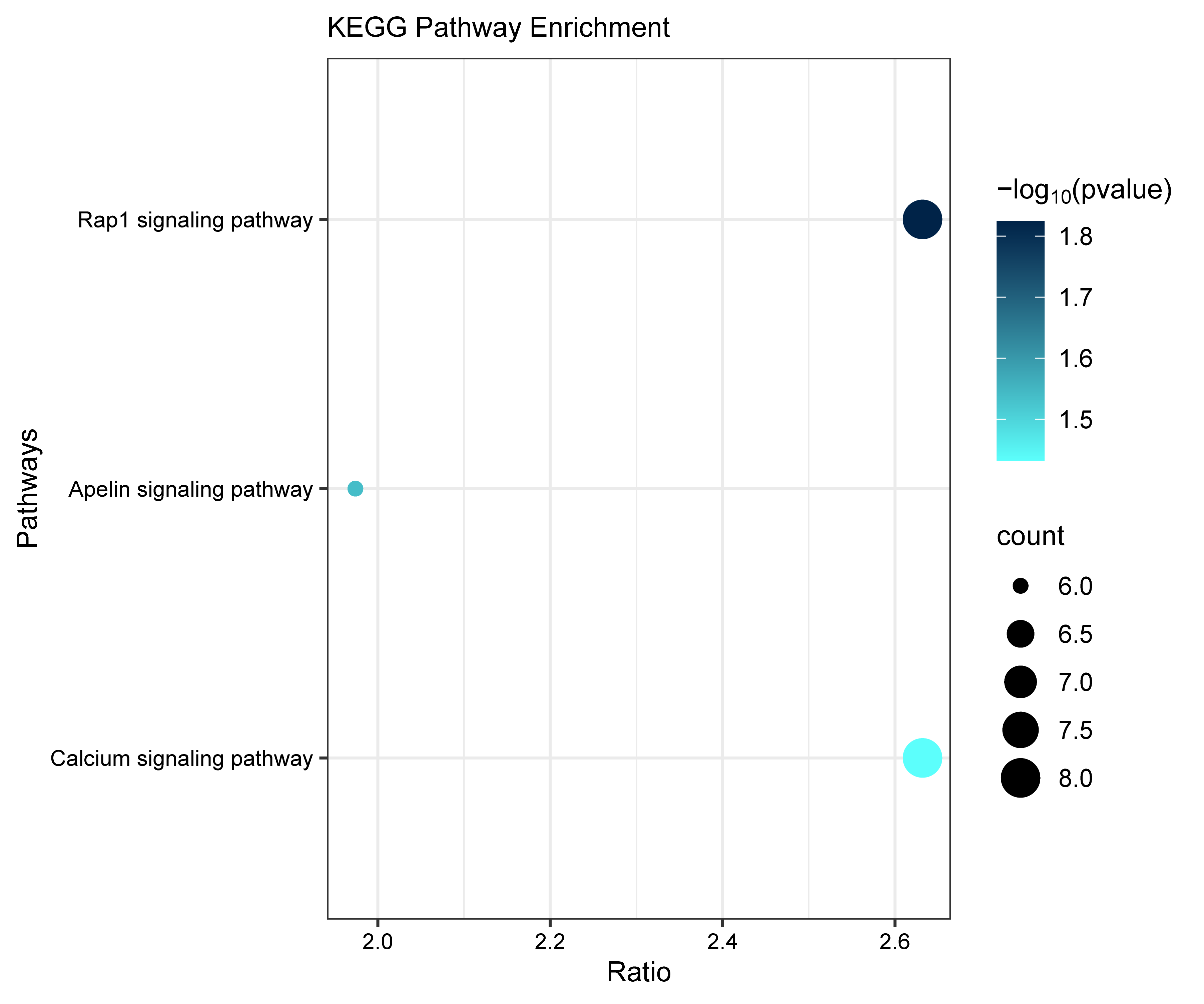

Supplement: Supplementary file 1 [file microorganisms-12-01170-s001.zip › Figure S12.tif]

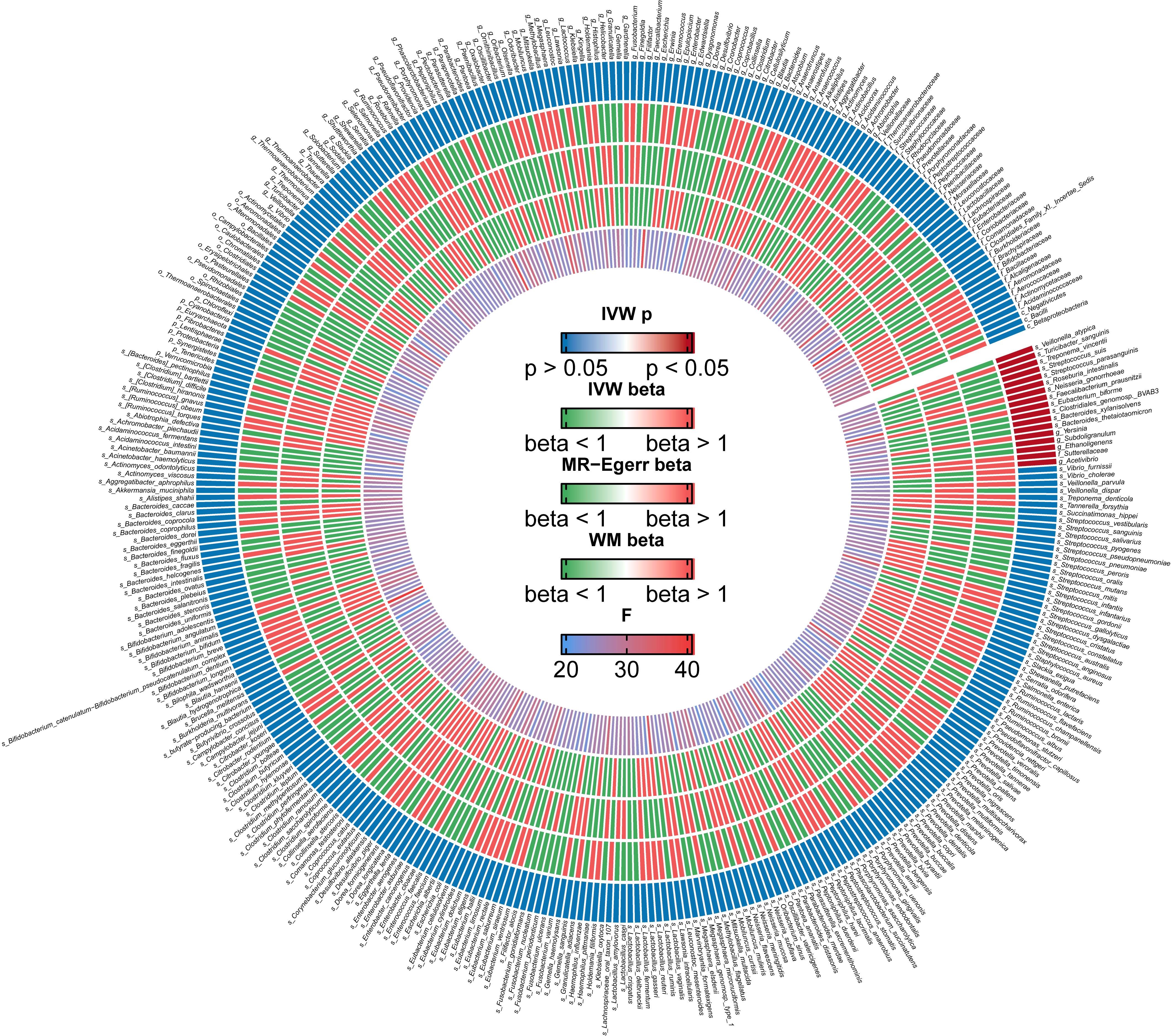

Supplement: Supplementary file 1 [file microorganisms-12-01170-s001.zip › Figure S5.tif]
